# Therapeutic potential of Jatimukuladi Varti Anjana in alkali burn-induced corneal injury and neovascularization in albino rats

**DOI:** 10.3389/fmed.2026.1921154

**Published:** 2026-09-16

**Authors:** Vishva S. Pandya, B. N. Ashwini, K. Sivabalaji

**Affiliations:** Department of Shalakya Tantra, Amrita School of Ayurveda, Amrita Vishwa Vidyapeetham, Amritapuri, India

**Keywords:** alkali burn, Anjana, anti-angiogenic, Ayurveda, corneal neovascularisation

## Abstract

**Introduction:**

Corneal transparency is maintained by its avascular nature, and disruption following injury can lead to corneal neovascularization (CNV), resulting in visual impairment. Although current treatment such as corticosteroids, NSAIDs, anti-VEGF agents, and surgical interventions are available, their use is often limited by adverse effects, recurrence, and cost. Jatimukuladdiv arti Anjana (JMV), a classical ayurvedic ophthalmic formulation with reported anti-inflammatory and tissue-healing properties, was evaluated for its therapeutic efficacy in an alkali burn-induced CNV model in albino rats.

**Methods:**

Eighteen Swiss albino rats (36 eyes) were used. Corneal injury was induced in albino rats using a standardized alkali burn model. Animals were allocated to control, standard treatment, and JMV-treated groups (*n* = 6 rats/group). Clinical assessments were performed at predetermined intervals using validated scoring criteria for corneal opacity, neovascularization, vessel size, corneal haze, and wound healing on days 0, 7 and 14 in a blinded manner. Histopathological analysis was conducted on day 15. In addition, liquid chromatography–mass spectrometry (LC–MS) analysis was performed to characterize the phytochemical profile of JMV.

**Results:**

JMV treatment was associated with significant improvement in corneal healing, characterized by reduced opacity, neovascularization, vascular enlargement, and corneal haze compared with the untreated control group. Histopathological findings supported these observations, revealing attenuation of inflammatory and fibrotic changes with improved tissue organization and epithelial restoration in treated animals.

**Discussion:**

The present study evaluated the therapeutic potential of Jatimukuladi Varti Anjana (JMV) in an experimental model of alkali burn-induced corneal injury and neovascularization in albino rats. The findings demonstrated that JMV significantly improved corneal healing, as evidenced by reduced corneal opacity, neovascularization, vessel size, and corneal haze compared with the untreated control group.

**Conclusion:**

JMV demonstrated significant anti-angiogenic and wound-healing potential in alkali-burn-induced CNV. These findings support its potential role as an integrative therapeutic option for corneal neovascularisation.

## Introduction

The cornea is a transparent avascular tissue that acts as a structural barrier and protects the eye against infections. Along with the tear film, it provides a proper anterior refractive surface for the eye. The cornea contributes to two-thirds of the refractive power of the eye (1).

The cornea and sclera constitute the outer covering of the eyeball. The main purpose of this coat is to protect structures inside the eye (1). The transparent cornea plays an essential role in vision by acting as both a mechanical barrier and a refractive surface. It consists of five layers, each with distinct functions, and its unique avascular (lack of blood vessels) nature is key to its transparency and proper functioning. Transparency and avascularity of the cornea are essential to preserve optimal vision as well as protect the eye against infection and structural damage (2).

Corneal transparency is maintained by the highly organized arrangement of collagen fibrils within the stromal layer, their uniform spacing, and the relative state of stromal dehydration regulated by the corneal endothelium. In addition, the absence of blood and lymphatic vessels minimizes light scattering, thereby preserving the optical clarity required for normal vision. Disruption of these structural and physiological mechanisms following injury or inflammation can result in corneal edema, loss of transparency, and impaired visual function (18).

Maintenance of the avascular state of the cornea has been termed ‘angiogenic privilege’ (2). This terminology parallels the special protection that the cornea enjoys against the immune rejection of grafted tissues, its ‘immune privilege.’ This designation implies that the absence of blood vessels in the corneal stroma is atypical, just as most parts of the body do not have special protection against immune rejection of foreign antigens (3). The causes of corneal neovascularisation, including persistent hypoxia, chronic inflammation, injury, and hereditary stem cell deficiency, have been thoroughly reviewed recently and are not discussed further (4).

Under specific pathological circumstances, new capillaries can grow within the cornea. Abnormally grown new vessels can invade the corneal stroma from pre-existing peri corneal structures as a result of a disruption in the balance of angiogenic and antiangiogenic factors that normally preserve corneal transparency and subsequently lead to corneal neovascularisation (4).

From an Ayurvedic perspective, the clinical manifestations of corneal neovascularization may be conceptually correlated with Sira Shukra based on similarities in vascular proliferation and corneal involvement. Acharya Vāgbhaṭa, in *Aṣṭāṅga Hṛdaya Uttarasthāna* (10/27), has elaborated *Sira Shukra* as a disease arising due to the vitiation of all three *doṣas* along with *Rakta doṣa*. This pathological process leads to the manifestation of abnormal vessels in the *Krishna Maṇḍala* (cornea), producing symptoms such as *Toda* (pricking pain), *Dāha* (burning sensation), and *Tāmra varṇa* (coppery discolouration). Classical descriptions suggest that *Sira Shukra* commonly develops as a complication of chronic corneal ulcers and can be correlated with conditions of modern ophthalmology, where persistent inflammation or ulceration results in corneal vascularisation (5).

In Ayurveda, *Anjana* is regarded as one of the most effective *Netrakriyākalpas* due to its simple administration, affordability, and suitability for long-term therapy (6). *Ropaṇa Anjana* is particularly significant in conditions associated with ulceration and vascularisation of the cornea, as its constituent herbs are known for their astringent, bitter, and cooling properties that support tissue healing. One such formulation is *JMV*, described in *Aṣṭāṅga Hṛdaya Uttarasthāna* (11/32). Prepared using *Jāti* (jasmine buds), *Lākṣā* (lac), *Gairika* (red ochre), and *Candana* (sandalwood), it is specifically indicated for pacifying *Pitta* and *Rakta doṣa*, and provides a basis for its therapeutic application in *Sira Śukra*, which closely parallels corneal neovascularisation described in modern ophthalmology (7).

Although the efficacy of Ayurvedic treatments for corneal neovascularisation has not been established through experimental or clinical studies, classical texts indicate their potential. *JMV* is traditionally described for conditions resembling corneal vascularisation and ulceration. Hence, the present study aims to evaluate its effect in an alkali burn–induced corneal neovascularisation model in albino rats to generate supportive experimental evidence.

## Materials and methods

### Study design

- Experimental, randomized, controlled animal study conducted in accordance with ARRIVE guidelines.

### Ethical approval

The study protocol was approved by the Institutional Animal Ethics Committee (IAEC), SDM Centre for Research in Ayurveda and Allied Sciences, Udupi (Approval No: SDMCRA/IAEC/AM-S-11) and conducted as per CPCSEA guidelines.

### Animals

- Eighteen healthy male Swiss albino rats (8–10 weeks old, 150–300 g) were used. Sample size was calculated using G*Power 3.1.9.7 software.
- The sample size was determined *a priori* using G*Power software based on a one-way ANOVA, assuming an effect size (f) of **0.90**, an *α* error probability of **0.05**, a statistical power (1−β) of **0.80**, and **three experimental groups**, resulting in a required total sample size of **18 animals (6 animals per group)**.

### Randomization and blinding

- Animals were randomly allocated into three groups (*n* = 6 per group). Clinical grading was performed by a masked observer.

### Preparation of the study drug—JMV

- The trial medicine JMV was prepared by a GMP-certified company.
- The classical Ayurvedic method of preparation of JMV was adopted for obtaining the study drug of 250 gm of JMV Jatimukula (bud of Jasminum grandiflorum), laksha (lacifier lacca), Chandana (Santalum album), and gairika (iron oxide). Each 50gm fine powder was collected, and 150 mL of distilled water was added. The powders were well mixed and made a fine paste, and rolled into the shape of varti. The obtained varti was dried under shade for 10–15 days and stored in an air-tight container (Table 1).
- Production certificate of JMV is attached.
- Physicochemical analysis, Quality control analysis and LCMS of JMV was done (certificates attached).

**Table 1:** Ingredients of JMV formulation with their botanical names, families and reported pharmacological activities.

SR no	Ingredients	Latin name	Family	Part	Ratio
1	Jatimukula	Jasminum grandiflorum	Oleaceae	Buds	1 part
2	Chandana	Santalum album	Santaleaceae	Wood powder	1 part
3	Laksha	Laccifer lacca	Kerriidae (Lac insects)	Purified powdered lac	1 part
4	Gairika	Red Oxide of iron	–	Sodhita Swarna gairika	1 part

### Rats’ maintenance

- All the animals used for the study were bred and maintained at the animal house of SDM Centre for Research in Ayurveda and Allied Sciences, Udupi, under the same environmental conditions, including location, light, temperature, food and other factors.
- 18 Swiss albino rats were selected and divided into 3 groups of 6 rats (*n* = 6) and kept in 3 cages. The animals were marked as head, neck, body, tail, and forelimbs, with no mark for the sample identification. The cages were labelled with study details (name of the researcher, group name, drug details, date of starting the experiment, etc.)

### Animal husbandry

Animal Husbandry: Animals were housed under standard laboratory conditions at a temperature of 21–24 °C, relative humidity of 57%–65%, and a 12-h light/dark cycle. They were maintained in polypropylene cages with sterilized paddy husk bedding and had free access to standard laboratory pellet diet and water *ad libitum*. The animals were acclimatized to laboratory conditions for five days prior to experimentation and monitored daily. Cages and accessories were cleaned and disinfected regularly to maintain hygienic conditions throughout the study.

### Inducing alkali-burn injury & compound treatment

1. Soak a round piece of filter paper, ∼3.5 mm in diameter, in a solution of 1 M NaOH (8).
2. Before administering the drug, the rats were individually put into an anaesthetic induction chamber with a volume of 8 L (20 cm × 20 cm × 20 cm). In the chamber, anaesthesia was induced with 1.9% Ethyl Ether (Diethyl ether) concentration, and their vital signs were monitored. The rats were watched for signs of sedation.

[Method used for Anaesthesia- Inhalation, chemical used- 1.9% Ethyl Ether (Diethyl ether)].

3. Using an eyedropper, topically apply a drop of 0.5% proparacaine hydrochloride to the corneal surface for local analgesia.
4. Using sterile forceps, pick up a piece of NaOH-soaked filter paper. Place the piece of NaOH-soaked filter paper on the central cornea. Leave it for 30 s to generate an acute alkali burn of ∼3.5 × 1.75 × 1.75 ℼr² in area. Note: Only one eye of the rat should be injured, with the other serving as a control.
5. Remove the filter paper. Using a 10 mL syringe, gently flush the eye with 10 mL of 1× PBS twice to wash away residual 1 M NaOH.

## Grouping

Thereafter, on the same day, the alkali-burned rats were randomly divided into 3 groups (6 rats—6 eyes/group): trial group, standard group, and control group. Trial group rats (*n* = 6 eyes) were treated with 0.6 mL/kg of JMV eye drops at 10 AM for 14 days. Standard group rats (*n* = 6 eyes) were treated with 0.108 mg/kg of 3% gentamicin sulphate eye drops at 10 AM for 14 days. The control group (*n* = 6 eyes) was left untreated for the baseline comparison. [The drug dose was derived as the animal equivalent dose (AED) from the human therapeutic dose using body weight conversion factors for Swiss albino rats].

Jatimukuladi Varti powder (200 g) was triturated with 150 mL of sterile rose water to prepare a suspension with a nominal concentration of approximately 1.33 g/mL. The preparation was filtered through sterile gauze before ocular administration, and the pH was approximately 6.4. The administered dose was calculated according to the body weight of each animal and instilled as 2–3 drops at 2–4-minute intervals.

## Clinical assessment criteria

On day 0,7 and day 14 of the trial, the clinical evaluation was conducted, and corneal neovascularisation was graded for opacity, vessel number, size, and haze. It was conducted by an Ayurvedic ophthalmologist in a blinded manner using standard grading criteria described below (9).

Performed an examination of the rat in a blinded fashion under a surgical microscope and scored corneal NV based on corneal opacity, NV, and vessel size.

1. Score corneal opacity on a scale of 0–4. 0 = completely clear; 1 = slightly hazy, iris and pupil easily visible; 2 = slightly opaque, iris and pupil still detectable; 3 = opaque, pupils hardly detectable; and 4 = completely opaque with no view of the pupil.
2. Score NV on a scale of 0–3. 0 = no neovessels; 1 = neovessels at the corneal limbus; 2 = neovessels spanning the corneal limbus and approaching the corneal center; 3 = neovessels spanning the corneal center.
3. Score vessel size on a scale of 0–3. 0 = no neovessels; 1 = neovessels detectable under surgical microscope; 2 = neovessels easily seen under surgical microscope; 3 = neovessels easily seen without the microscope.

On day 15, the rats were euthanised, and the corneal tissues were carefully harvested, and the tissues were sent for Hematoxylin & Eosin Staining (*n* = 2 eyes/ group).

For histopathological evaluation, two treated eyes from each experimental group were randomly selected in a blinded manner. The limited number of samples was chosen in accordance with the approved IAEC protocol and the principles of the 3Rs to minimize animal use while obtaining representative histological findings.

### Method of euthanasia

On day 15, the rats were euthanised in accordance with CPCSEA and AVMA guidelines. Euthanasia was performed using gradual CO₂ inhalation in a dedicated chamber, followed by cervical dislocation (for rats <200 g) or bilateral thoracotomy (for larger rats) to ensure death. This method is widely accepted as humane and reproducible for laboratory rodents.

Corneal dissection and fixation: Enucleated eyes are fixed in 4% solution of paraformaldehyde (PFA) at 4 °C, then transferred to PBS for removal of excess tissue. Using a surgical microscope, the eye is perforated near the cornea to release fluid, and the anterior portion (cornea) is separated from the posterior eye and post-fixed in 4% PFA overnight at 4 °C.

## Statistical analysis

Statistical analysis was performed using the Graphpad software followed by Kruskal–Wallis test (non-parametric ANOVA) to compare differences among groups.

## Results

### Inter-group comparison of corneal neovascularization parameters

#### Effect of JMV on corneal opacity (parameter 1)

The data related to the effect of the trial drug on Corneal Opacity at day 0 in the alkali burn injury rat eye has been depicted in Table 2.

**Table 2:** Effect of trial drug on corneal opacity at day 0.

Group	Corneal opacity	% change
Contol	0.33 ± 0.21	–
Standard	0.33 ± 0.21	0
Trial	0.16 ± 0.16*	51.51↓

DATA: MEAN ± SEM.

* *P* < 0.05.

The mild opacity scores recorded on Day 0 most likely represent acute corneal edema and early stromal haze immediately following alkali injury rather than true corneal opacity. Since permanent structural changes had not yet developed, the minor baseline differences are likely due to subjective clinical grading of the acute inflammatory response.

The data related to the effect of the trial drug on Corneal Opacity at day 7 in the alkali burn injury rat eye has been depicted in Table 3.

**Table 3:** Effect of trial drug on corneal opacity at day 7.

Group	Corneal opacity	% change
Contol	3.83 ± 0.166	–
Standard	3.83 ± 0.166	0
Trial	2.33 ± 0.21*	39.16↓

DATA: MEAN ± SEM.

* *P* < 0.01.

On Day 7, the mean corneal opacity score was 3.83 ± 0.166 in the standard group and 2.33 ± 0.21 in the trial group. The mean difference between the groups was 1.50, and the reduction observed in the trial group was statistically highly significant (*P* < 0.01).

The data related to the effect of the trial drug on Corneal Opacity at day 14 in the alkali burn injury rat eye has been depicted in Table 4.

**Table 4:** Effect of trial drug on corneal opacity at day 14.

Group	Corneal opacity	% change
Contol	2.66 ± 0.33	–
Standard	2.33 ± 0.49	12.40↓
Trial	1.66 ± 0.21	37.59↓

DATA: MEAN ± SEM

*P* > 0.05

On Day 14, the mean corneal opacity score was 2.33 ± 0.49 in the standard group and 1.66 ± 0.21 in the trial group. The mean difference between the groups was 0.67, with the trial group showing a comparatively lower corneal opacity score. However, the observed difference was not statistically significant (*P* > 0.05).

#### Effect of JMV on neo-vessel formation (parameter 2)

The data related to the effect of the trial drug on Neo-vessels at day 0 in the alkali burn injury rat eye has been depicted in Table 5.

**Table 5:** Effect of trial drug on neo-vessel formation at day 0.

Group	Neo vessels	% change
Contol	0 ± 0.00	–
Standard	0 ± 0.00	0
Trial	0 ± 0.00	0

DATA: MEAN ± SEM.

No changes in Neo vessels on day 0 in the standard and trial group, when compared to the control group, shows Corneal neovascularization typically takes several days to develop post-injury. On Day 0, no new vessels are expected yet.

The data related to the effect of the trial drug on Neo-vessels at day 7 in the alkali burn injury rat eye has been depicted in Table 6.

**Table 6:** Effect of trial drug on neo-vessel formation at day 7.

Group	Neo vessels	% change
Contol	2 ± 0.25	–
Standard	1.83 ± 0.166	8.5↓
Trial	2.33 ± 0.21	16.5↑

DATA: MEAN ± SEM.

*P* > 0.05.

On Day 14, the mean neovessel score was 1.83 ± 0.166 in the standard group and 2.33 ± 0.21 in the trial group. The trial group exhibited a marginally higher neovessel score, with a mean difference of 0.50. However, this difference was not statistically significant (*P* > 0.05)

The data related to the effect of the trial drug on Neo-vessels at day 14 in the alkali burn injury rat eye has been depicted in Table 7.

**Table 7:** Effect of trial drug on neo-vessel formation at day 14.

Group	Neo vessels	% change
Contol	2.33 ± 0.33	–
Standard	2.16 ± 0.47	7.29↓
Trial	1.33 ± 0.49	42.92↓

DATA: MEAN ± SEM.

*P* > 0.05.

On Day 14, the mean neovessel score was 2.16 ± 0.47 in the standard group and 1.33 ± 0.49 in the trial group. The mean difference between the groups was 0.83, with the trial group exhibiting a comparatively lower neovessel score. However, this difference was not statistically significant (*P* > 0.05).

#### Effect of JMV on vessel size

The data related to the effect of the trial drug on Vessel size at day 0 in the alkali burn injury rat eye has been depicted in Table 8.

**Table 8:** Effect of trial drug on vessel size at day 0.

Group	Vessel size	% change
Contol	0 ± 0.00	–
Standard	0 ± 0.00	0
Trial	0 ± 0.00	0

DATA: MEAN ± SEM.

No changes in Neo vessels and its size on day 0 in the standard and trial group, when compared to the control group, shows Corneal neovascularization typically takes several days to develop post-injury. On Day 0, no new vessels are expected yet.

The data related to the effect of the trial drug on Vessel size at day 7 in the alkali burn injury rat eye has been depicted in Table 9.

**Table 9:** Effect of trial drug on vessel size at day 7.

Group	Vessel size	% change
Contol	1.33 ± 0.21	–
Standard	1.16 ± 0.30	12.78↓
Trial	1.33 ± 0.33	0

DATA: MEAN ± SEM.

*P* > 0.05.

The mean vessel size score on Day 14 was 1.16 ± 0.30 in the standard group and 1.33 ± 0.33 in the trial group. The mean difference between the two groups was 0.17. However, the observed difference between the standard and trial groups was not statistically significant (*P* > 0.05).

The data related to the effect of the trial drug on Vessel size at day 14 in the alkali burn injury rat eye has been depicted in Table 10.

**Table 10:** Effect of trial drug on vessel size at day 14.

Group	Vessel size	% change
Contol	2.66 ± 0.33	–
Standard	2.33 ± 0.49	12.40↓
Trial	1.66 ± 0.21	37.59↓

DATA: MEAN ± SEM.

*P* > 0.05.

The mean vessel size score on Day 14 was 2.33 ± 0.49 in the standard group and 1.66 ± 0.21 in the trial group. The trial group demonstrated a lower mean vessel size score than the standard group, with a mean difference of 0.67. However, the observed difference between the two groups was not statistically significant (*P* > 0.05).

### Intra-group comparison of corneal neovascularization parameters

Day 0 scores represent the immediate post-burn corneal appearance (predominantly acute stromal edema and early haze) rather than established corneal opacity. Corneal opacity scores increased significantly in both the standard and trial groups on Day 7 compared with baseline (*P* < 0.01). A subsequent reduction in opacity scores was observed on Day 14, suggesting gradual restoration of corneal clarity and recovery from alkali burn-induced damage (Table 11).

**Table 11:** Intra-group comparison of corneal opacity scores.

Group	Day 0	Day 7	Day 14	*P* value
Standard	0.33 ± 0.21	3.83 ± 0.16*	2.33 ± 0.49	*P* < 0.01
Trial	1.66 ± 1.66	2.33 ± 0.21*	1.66 ± 0.21	*P* < 0.01

The symbol “*” indicate the statistical significance of the corresponding mean ± SEM values.

Intra-group analysis of neovessel formation scores showed an increase in vascularization on Day 7 in both the standard and trial groups compared with baseline. The increase was highly significant in the trial group (*P* < 0.01), while a significant reduction in neovessel scores was observed by Day 14, indicating regression of corneal neovascularization and progressive healing following treatment (Table 12).

**Table 12:** Intra-group comparison of neovessel scores.

Group	Day 0	Day 7	Day 14	*P* value
Standard	0 ± 0.00	1.83 ± 0.16	2.16 ± 0.47*	*P* < 0.05
Trial	0 ± 0.00	2.33 ± 0.21**	1.66 ± 0.21	*P* < 0.01

The symbol “*” indicate the statistical significance of the corresponding mean ± SEM values, while “**” denotes very statistically significant.

Intra-group analysis of vessel size scores demonstrated an increase following alkali burn injury, with values peaking on Day 7 in both treatment groups. The increase was statistically significant in the trial group (*P* < 0.05), whereas changes in the standard group remained non-significant (*P* > 0.05). A mild reduction in vessel size was observed by Day 14, indicating partial regression of vascular enlargement and ongoing corneal recovery (Table 13).

**Table 13:** Intra-group comparison of vessel size scores.

Group	Day 0	Day 7	Day 14	*P* value
Standard	0 ± 0.00	1.16 ± 0.30	1.33 ± 0.33	*P* > 0.05
Trial	0 ± 0.00	1.33 ± 0.33*	1.16 ± 0.30	*P* < 0.05

The symbol “*” indicate the statistical significance of the corresponding mean ± SEM values.

Intra-group analysis of corneal haze scores demonstrated a significant increase on Day 7 in both the standard and trial groups compared with baseline, reflecting the acute effects of alkali burn injury. A subsequent reduction in haze scores was observed by Day 14, indicating progressive restoration of corneal transparency and recovery following treatment (Table 14).

**Table 14:** Intra-group comparison of corneal haze scores.

Group	Day 0	Day 7	Day 14	*P* value
Standard	0 ± 0.00	3.50 ± 0.22**	2.50 ± 0.56	*P* < 0.01
Trial	0 ± 0.00	2.33 ± 0.61*	1.66 ± 0.49	*P* < 0.05

The symbol “*” indicate the statistical significance of the corresponding mean ± SEM values, while “**” denotes very statistically significant.

### Assessment of corneal wound healing/re-epithelialization

The effect of the trial drug on corneal wound healing and re-epithelialization assessed by fluorescein staining at Day 14 is presented in Table 15.

**Table 15:** Effect of JMV on corneal re-epithelialization.

Group	Corneal wound healing/re-epithelialization	% change
Contol	1.66 ± 0.54	–
Standard	1.66 ± 0.49	0
Trial	1.66 ± 0.47	0

DATA: MEAN ± SEM.

*P* > 0.05.

The mean corneal wound healing/re-epithelialization score was comparable among the control, standard, and trial groups on Day 14, the trial drug JMV was found to be as effective as the standard drug Gentamycin with comparable efficacy in promoting corneal wound healing and re-epithelialization.

#### Histopathological observations (H&E staining)

Ulcer-Black arrow, Epithelial hyperplasia- Blue-arrow, Inflammatory infiltrate- Red arrow, Fibrosis- Green arrow, Haemorrhage-purple arrow (Figures 1–3).

**Figure 1:**
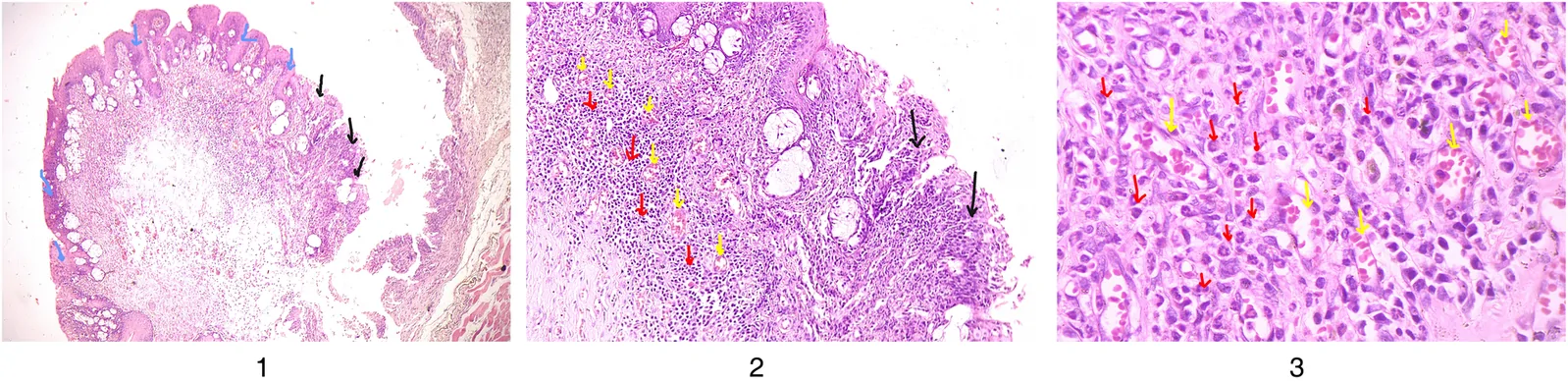
Control group: histopathological sections showed 40x-epithelial hyperplasia with exophytic and endophytic growth and formation of retepegs with ulcer (1). 100x-areas showing ulcerative epithelium, inflammatory infiltrate in stroma (2). 400x-neutrophilic infiltrate, neovascularisation in the stroma (3) (H&E stain).

**Figure 2:**
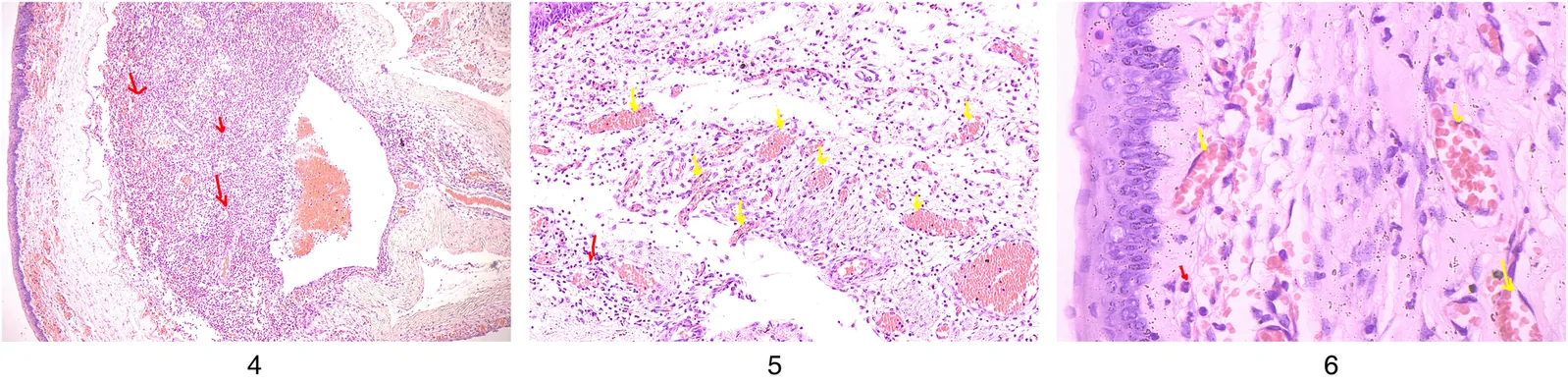
Standard group: histopathological sections showed 40x-neovascularisation seen with reduced inflammation in corneal stroma. inflammation seen in the anterior chamber region (4). 100x-stromal region shows neovascularisation with reduced inflammatory infiltration (5). 400x-reduced histological changes in cornea region (6) (H&E stain).

**Figure 3:**
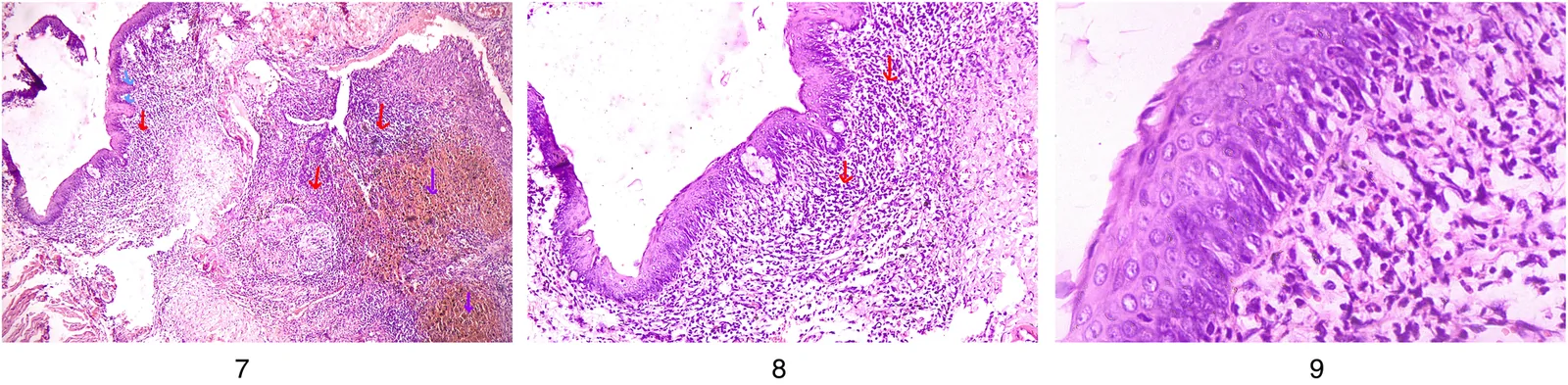
Trial group: histopathological sections showed 40x-reduced epithelial hyperplasia compared with ctl. formation of retepegs seen (7). 100x-reduced epidermal hyperplasia compared with ctl. (8). 400x-reduced epidermal hyperplasia compared with ctl (9) (H&E stain).

## Discussion

### LCMS analysis of JMV

LC–MS analysis of Jatimukuladi Varti Anjana revealed a chemically diverse phytoconstituent profile comprising 867 compounds in the polar fraction and 192 compounds in the non-polar fraction. Among the identified metabolites, several pharmacologically important compounds were detected, including **Luteolin, Genistein,** Fusidic acid, Aleuritic acid, Iriomoteolide 1a, Sinapinic acid, Palmitoleic acid, 3,4-Dihydroxyphenylpropionic acid, 16-Hydroxyhexadecanoic acid, 2-Hydroxymyristic acid, 3-Hydroxymyristic acid, 9,10-Dihydroxystearic acid, 12-Hydroxystearic acid (12-HSA), Ambrettolide and Hydroquinone, along with several glycosides, steroidal derivatives and fatty acid esters. These compounds belong to diverse chemical classes such as flavonoids, phenolic acids, fatty acids, hydroxy fatty acids, lactones, steroidal compounds and other secondary metabolites, indicating the complex phytochemical composition of the formulation (Figure 4).

**Figure 4:**
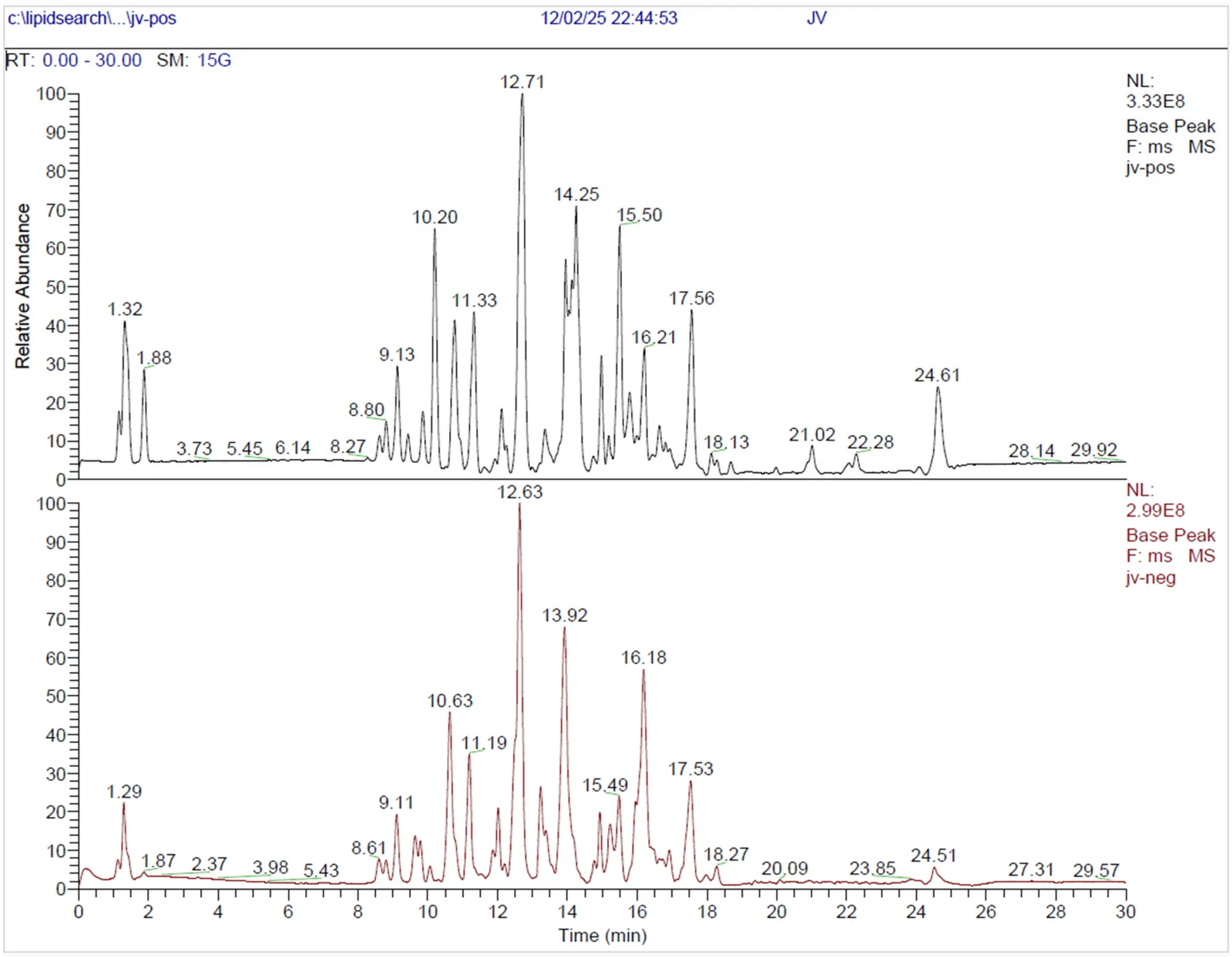
LC-MS analysis of JMV.

### Jatimukuladi Varti Anjana as an anti-neovascular agent

**Joussen et al.** (11) evaluated the anti-angiogenic effects of dietary **flavonoids and iso-flavonoids** in a rabbit model of basic fibroblast growth factor (bFGF)-induced corneal neovascularization (10, 11). The study demonstrated that topical administration of Genistein and **Luteolin** significantly inhibited the growth of newly formed corneal blood vessels compared with untreated controls. The authors suggested that **Genistein** exerted its anti-angiogenic effect primarily through inhibition of protein tyrosine kinase-mediated VEGF signalling and endothelial cell proliferation, whereas Luteolin suppressed angiogenesis by inhibiting VEGF expression and inflammatory pathways, including **NF-κB and COX-2.** These findings indicate that both phytoconstituents possess potent anti-neovascular properties and may act synergistically to suppress pathological corneal angiogenesis. Since both Genistein and Luteolin were identified in the LC–MS analysis of Jatimukuladi Varti, their presence may contribute to the reduction in corneal neovascularization observed in the present study by inhibiting endothelial cell proliferation, migration, and angiogenic signalling.

### Jatimukuladi Varti as an anti-proliferative agent

LC–MS analysis of **Jatimukuladi Varti** identified **Genistein, Luteolin and Iriomoteolide 1a**, all of which have reported anti-proliferative properties (12, 13). **Genistein**, a natural isoflavone and protein tyrosine kinase inhibitor, suppresses cell proliferation by inducing cell-cycle arrest, inhibiting **PI3 K/Akt** and **MAPK** signalling, promoting apoptosis and downregulating **VEGF-mediated endothelial proliferation** (Tuli et al.). These actions may inhibit endothelial cell and stromal fibroblast proliferation, thereby reducing corneal neovascularization and fibrosis.

Similarly, **Luteolin** inhibits abnormal cell growth by suppressing **PI3 K/Akt, ERK, STAT3 and mTOR** signalling pathways, resulting in cell-cycle arrest, reduced endothelial proliferation and decreased expression of **VEGF** and matrix metalloproteinases (Imran et al.; Tuli et al.). These mechanisms may contribute to limiting pathological tissue remodelling following alkali burn.

Collectively, these bioactive constituents suggest that **Jatimukuladi Varti** exerts anti-proliferative effects through inhibition of **protein tyrosine kinase, PI3 K/Akt, MAPK, STAT3 and VEGF** signalling pathways.

### Jatimukuladi Varti as an anti-inflammatory agent

LC–MS analysis of **Jatimukuladi Varti** identified **Luteolin, Genistein, Sinapinic acid, 3,4-Dihydroxyphenylpropionic acid** and **Palmitoleic acid**, all of which possess well-documented anti-inflammatory properties (10, 14–16). **Luteolin** suppresses **NF-κB** activation, inhibits **COX-2** and **iNOS**, and reduces the production of pro-inflammatory cytokines such as **TNF-α, IL-1β and IL-6** (11). Similarly, **Genistein** inhibits **NF-κB** and **MAPK** signalling, attenuates leukocyte recruitment and decreases inflammatory cytokine expression, thereby indirectly suppressing inflammation-induced **VEGF** production (14). These mechanisms may have contributed to the reduction in corneal inflammation and congestion observed in the present study.

Additionally, **Sinapinic acid** inhibits the **NLRP3 inflammasome**, suppresses **NF-κB** signalling and reduces oxidative stress, thereby limiting inflammatory tissue injury (15). **3,4-Dihydroxyphenylpropionic acid** further supports this effect by scavenging reactive oxygen species and suppressing **NF-κB-mediated** cytokine production, while **Palmitoleic acid** has been shown to reduce **TNF-α, IL-1β and IL-6** and promote tissue repair (19). Collectively, these phytoconstituents modulate multiple inflammatory pathways, including **NF-κB, COX-2, MAPK and NLRP3 inflammasome signalling**, thereby reducing inflammatory cell infiltration and creating a favourable environment for corneal healing. Their synergistic actions may explain the decreased inflammatory changes and subsequent inhibition of corneal neovascularization observed in the present study.

### Additional therapeutic effects of Jatimukuladi Varti

#### Anti-microbial activity

LC–MS analysis of Jatimukuladi Varti identified **Fusidic acid, 2-Hydroxymyristic acid and 3-Hydroxymyristic acid**, indicating that the formulation contains constituents with potential antimicrobial activity (17).

Among these compounds, **Fusidic acid** is a steroidal antibiotic that has been widely used in ophthalmology for the treatment of superficial bacterial eye infections, particularly those caused by Staphylococcus aureus, including methicillin-resistant strains (MRSA). **Godtfredsen et al**. first demonstrated that fusidic acid inhibits bacterial protein synthesis by specifically binding to **elongation factor G (EF-G),** thereby preventing translocation during peptide chain elongation. This unique mechanism effectively suppresses bacterial growth while exhibiting excellent activity against Gram-positive cocci. Because of its prolonged retention on the ocular surface and good corneal penetration, topical fusidic acid has become an established treatment for bacterial conjunctivitis and blepharitis. By preventing bacterial colonization of the injured cornea, fusidic acid may reduce secondary inflammation and provide a favorable environment for epithelial regeneration and stromal repair.

#### Anti-oxidant activity

LC–MS analysis of **Jatimukuladi Varti** identified several phytoconstituents with established antioxidant properties, including **Luteolin, Genistein, Sinapinic acid, 3,4-Dihydroxyphenylpropionic acid** and **Palmitoleic acid**. **Luteolin** and **Genistein** have been reported to scavenge reactive oxygen species (ROS), enhance endogenous antioxidant defence systems and suppress oxidative stress-induced **NF-κB** signalling, thereby protecting cells from oxidative injury (Seelinger et al.; Tuli et al.) (10). Similarly, **Sinapinic acid** activates the **Nrf2/HO-1** antioxidant pathway, inhibits lipid peroxidation and attenuates oxidative tissue damage, while **3,4-Dihydroxyphenylpropionic acid** exerts potent free radical scavenging activity and protects cells against ROS-mediated injury. **Palmitoleic acid** further contributes through its antioxidant and cytoprotective effects by reducing oxidative stress and promoting tissue repair. Collectively, these bioactive constituents may mitigate oxidative damage during the early phase of alkali burn, suppress downstream inflammatory and angiogenic signalling, preserve corneal epithelial and stromal integrity, and facilitate physiological wound healing. The synergistic antioxidant activity of these compounds may therefore have contributed to the reduction in corneal opacity, inflammation and neovascularization observed in the present study.

Taken together, the therapeutic effect of **Jatimukuladi Varti** is likely to result from the synergistic interaction of its multiple bioactive constituents rather than the action of a single compound. By simultaneously attenuating oxidative stress, suppressing inflammatory cytokines, inhibiting VEGF-dependent angiogenesis, regulating stromal fibrosis, promoting epithelial regeneration and reducing the risk of secondary microbial infection, the formulation addresses the major pathological events responsible for alkali burn-induced corneal neovascularization. This multitarget mode of action provides a plausible scientific explanation for the significant reduction in corneal neovascularization, corneal opacity and inflammatory changes, together with the enhanced corneal healing observed in the treatment groups of the present study.

### Limitation of the study

A limitation of the present study is the absence of a vehicle-control group receiving the formulation base without active ingredients. Inclusion of such a group in future studies would help distinguish the specific pharmacological effects of Jatimukuladi Varti Anjana from any potential influence of the vehicle.

The use of gentamicin as the standard comparator represents a limitation of the present study, as it primarily provides antimicrobial protection rather than direct anti-angiogenic activity. Future studies should compare JMV with established therapies such as topical corticosteroids or anti-VEGF agents to further define its therapeutic role in corneal neovascularization.

The osmolarity of the prepared ophthalmic formulation was not evaluated. Future studies should include detailed physicochemical characterization to further establish ocular compatibility and formulation quality.

Histopathological assessment was performed on a limited number of representative samples (*n* = 2 eyes/group) in accordance with the approved ethical protocol and the 3Rs principle. Although the findings supported the clinical observations, future studies including a larger number of histological samples would provide stronger pathological validation.

Histopathological evaluation was qualitative rather than quantitative. Future investigations incorporating standardized semi-quantitative scoring systems or digital image analysis for epithelial thickness, inflammatory cell infiltration, stromal changes, and neovascular density would provide more objective pathological validation of the therapeutic effects of Jatimukuladi Varti Anjana.

A limitation of the present study is the use of diethyl ether for anesthesia, which was selected according to the approved institutional protocol and available facilities at the time of the study. Future studies should consider the use of inhalational anesthetics such as isoflurane in accordance with contemporary laboratory animal welfare recommendations.

Detailed procedural parameters for CO₂ euthanasia (e.g., flow rate and chamber fill method) were not documented during the study and therefore could not be reported retrospectively.

## Conclusion

In summary, this experimental evaluation provides the first preclinical evidence supporting the efficacy of Jātīmukulādi Varti Anjana in corneal neovascularization. The statistically significant improvements in opacity, vessel size, vascular regression, and wound healing reinforce its potential as a therapeutic option. However, further studies, including mechanistic experiments and clinical trials, are warranted to validate these findings and establish its role in integrative ophthalmic care.

## Data Availability

The original contributions presented in the study are included in the article/Supplementary Material, further inquiries can be directed to the corresponding author.
