# Correction: Therapeutic potential of Jatimukuladi Varti Anjana in alkali burn-induced corneal injury and neovascularization in albino rats

**DOI:** 10.3389/fmed.2026.2023065

**Published:** 2026-10-19

**Authors:** Vishva S. Pandya, B. N. Ashwini, K. Sivabalaji

**Affiliations:** Department of Shalakya Tantra, Amrita School of Ayurveda, Amrita Vishwa Vidyapeetham, Amritapuri, India

**Keywords:** alkali burn, Anjana, anti-angiogenic, Ayurveda, corneal neovascularisation

Author Vishva S. Pandya was erroneously included as corresponding author. The correct corresponding author is Sivabalaji K.

The Corresponding author's email address should also be updated to: dr.sivabalaji@ay.amrita.edu

The original version of this article has been updated.

